# Intentional Weight Loss and Longevity in Overweight Patients with Type 2 Diabetes: A Population-Based Cohort Study

**DOI:** 10.1371/journal.pone.0146889

**Published:** 2016-01-25

**Authors:** Rasmus Køster-Rasmussen, Mette Kildevæld Simonsen, Volkert Siersma, Jan Erik Henriksen, Berit Lilienthal Heitmann, Niels de Fine Olivarius

**Affiliations:** 1 The Research Unit for General Practice and Section of General Practice, Department of Public Health, University of Copenhagen, Copenhagen, Denmark; 2 Department of Clinical Research, University of Southern Denmark, Odense, Denmark; 3 Finsen Center, Rigshospitalet, Copenhagen, Denmark; 4 Department of Endocrinology, Odense University Hospital, Odense, Denmark; 5 Research Unit for Dietary Studies, The Parker Institute and Institute of Preventive Medicine, Capital Region, Bispebjerg and Frederiksberg University Hospitals, Frederiksberg, Denmark; 6 The Boden Institute of Obesity, Nutrition, Exercise & Eating Disorders, University of Sydney, Sydney, Australia; 7 The National Institute of Public Health, University of Southern Denmark, Copenhagen, Denmark; University of Catanzaro Magna Graecia, ITALY

## Abstract

**Objective:**

This study examined the influence of weight loss on long-term morbidity and mortality in overweight (BMI≥25kg/m^2^) patients with type 2 diabetes, and tested the hypothesis that therapeutic intentional weight loss supervised by a medical doctor prolongs life and reduces the risk for cardiovascular disease in these patients.

**Methods:**

This is a 19 year cohort study of patients in the intervention arm of the randomized clinical trial Diabetes Care in General Practice. Weight and prospective intentions for weight loss were monitored every third month for six years in 761 consecutive patients (≥40 years) newly diagnosed with diabetes in general practices throughout Denmark in 1989–92. Multivariable Cox regression was used to estimate the association between weight change during the monitoring period (year 0 to 6) and the outcomes during the succeeding 13 years (year 6 to 19) in 444 patients who were overweight at diagnosis and alive at the end of the monitoring period (year 6). The analysis was adjusted for age, sex, education, BMI at diagnosis, change in smoking, change in physical activity, change in medication, and the Charlson comorbidity 6-year score. Outcomes were from national registers.

**Results:**

Overall, weight loss regardless of intention was an independent risk factor for increased all-cause mortality (P<0.01). The adjusted hazard ratio for all-cause mortality, cardiovascular mortality, and cardiovascular morbidity attributable to an intentional weight loss of 1 kg/year was 1.20 (95%CI 0.97–1.50, P = 0.10), 1.26 (0.93–1.72, P = 0.14), and 1.06 (0.79–1.42, P = 0.71), respectively. Limiting the analysis to include only those patients who survived the first 2 years after the monitoring period did not substantially change these estimates. A non-linear spline estimate indicated a V-like association between weight change and all-cause mortality, suggesting the best prognosis for those who maintained their weight.

**Conclusions:**

In this population-based cohort of overweight patients with type 2 diabetes, successful therapeutic intentional weight loss, supervised by a doctor over six years, was not associated with reduced all-cause mortality or cardiovascular morbidity/mortality during the succeeding 13 years.

## Introduction

Weight loss is recognized as an important first-line treatment of overweight individuals (BMI ≥25 kg/m^2^) with type 2 diabetes. Losing weight has a well-documented short-term positive effect on intermediate outcomes such as glycaemic control, blood pressure, and dyslipidemia [1–5]. Indeed, weight loss is regarded by many clinicians as an effective secondary prevention for cardiovascular disease in overweight patients with diabetes. This view is not only supported by the favorable clinical effects of weight loss on risk factors, but also by results from cohort studies, suggesting that intentional weight loss reduces mortality in these patients [6–12]. Nevertheless, the large randomized Look AHEAD trial was unable to confirm the health benefits related to weight loss, and Look AHEAD was stopped prematurely after 9.6 years of intervention with diet and exercise [13]. Results from Look AHEAD showed, that despite a greater weight loss in the intervention compared with the control group, neither mortality nor cardiovascular morbidity was reduced. The participants in Look AHEAD were a selection of relatively healthy overweight adults with prevalent type 2 diabetes, and the authors stated that the results could not be generalized, as the participants in Look AHEAD were not representative of the background population of patients with type 2 diabetes [13].

In contrast to the Look AHEAD study, the randomized clinical trial, Diabetes Care in General Practice (DCGP), included a population-based sample of consecutive patients newly diagnosed with type 2 diabetes in 1989–92. The participants were representative of the background population with incident type 2 diabetes 25 years ago in a Northern European setting. This paper is a cohort study of the group of overweight patients in the well-monitored intervention arm of DCGP. Unintentional weight loss, caused by unrecognized fatal disease (pathological weight loss) is an important bias when studying the association between therapeutic weight loss and survival [11,12,14,15]. Therefore, our primary interest was to examine weight changes among patients who reported an intended aim to lose weight. The objective was to estimate the long-term all-cause mortality, cardiovascular mortality, and cardiovascular morbidity risk attributable to weight change in a population-based sample of overweight patients with newly diagnosed type 2 diabetes. Our hypothesis was that therapeutic intentional weight loss, supervised by a medical doctor, improves health and prolongs life in these patients.

## Materials and Methods

### The study design at a glance

**Fig 1:**
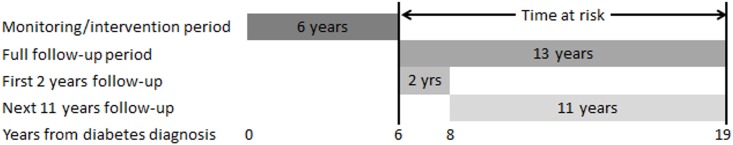
Timeline of the cohort study. Patients, newly diagnosed with diabetes, were included at year 0. The exposure of interest was weight change during year 0–6 (the monitoring period). Only patients surviving the monitoring period were included in the present analyses. The follow-up period was 13 years. The hazard ratios (HR) for mortality and morbidity were also estimated separately for the first 2 years of follow-up (year 6–8) and for the remaining 11 years (year 8–19), as bias from pathological weight loss was expected to be greater during the first two years of follow-up.

The timeline and basic design of this cohort study of overweight participants in the intervention arm of the DCGP trial is explained in Fig 1. The study is designed to minimize bias from pathological weight loss. The DCGP intervention consisted of structured care and individualized goal setting for risk factors and weight loss [5,16]. Approximately half of the overweight patients chose weight loss as one of their individual goals for managing their disease throughout the 6 years of intervention. We categorized this group as patients with an ‘intention to lose weight’. The associations between weight change and later morbidity or mortality were analyzed separately for patients with the ‘intention to lose weight’ and patients with the ‘intention to maintain weight’, and the main analysis in this cohort study was the relation between weight change and subsequent mortality or morbidity in the group of patients with the ‘intention to lose weight’.

### The DCGP study

**Fig 2:**
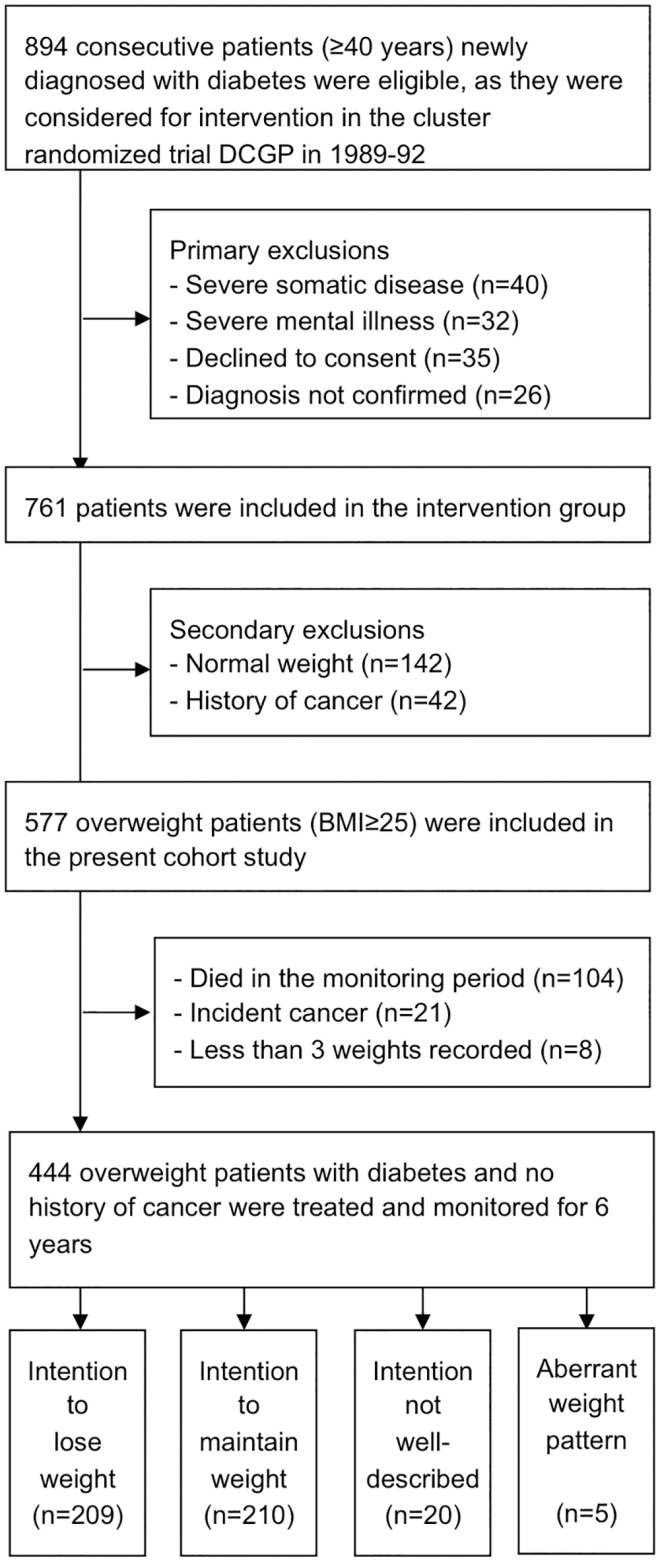
Patient flow.

DCGP included 1381 consecutive patients newly diagnosed with type 2 diabetes in general practices throughout Denmark (Fig 2). The patients were aged 40 years or older. The family physicians/general practitioners (GPs) were randomized to give either routine care or the intervention: structured personal care [5,16]. The intervention aimed at managing glycaemia, blood pressures, total cholesterol, triglycerides, and unhealthy lifestyle by means of individualized goal setting. The personal goals were set in an agreement between the doctor and the patient, and the goals were continuously adjusted. Altogether, 761 patients were included in the intervention arm, and every third month, over a period of 6 years, patients were invited to check-ups with their GP. Among other measures, body weight and a goal for weight change during the next 3 months were recorded at each visit. For overweight patients, the doctors in the study were encouraged to reach an agreement with patients on obtaining small and realistic weight reduction, recording it, and following it up at the next check-up. It was suggested to the intervention doctors that they recommend increased physical activity and simple dietary changes to patients. Dietary changes included eating 5–6 meals per day, an increase in the intake of complex carbohydrate to at least 50% of the diet, and, in particular, an increase in the intake of water soluble fibers, a reduction in alcohol, and restricting fat intake to a maximum of 30% [5]. The doctors in the intervention group were supported by clinical guidelines and continuous medical education. The recommended management of patients did not differ according to the patients’ level of overweight. Both at diabetes diagnosis and after 6 years of intervention there was no statistically significant difference in body weight between the two randomization arms [5].

### Participants

The present cohort study included the 444 overweight patients from the DCGP intervention arm (BMI at diagnosis ≥25 kg/m2) who were alive after 6 years of intervention, and who had at least 3 valid measurements of weight (Fig 2). The patients in the control arm were not included in the present study, as their body weight and intentions were not monitored every three months. Patients diagnosed with cancer (not including benign skin cancers) at any time before or during the monitoring period were excluded.

### Definition of weight change

**Fig 3:**
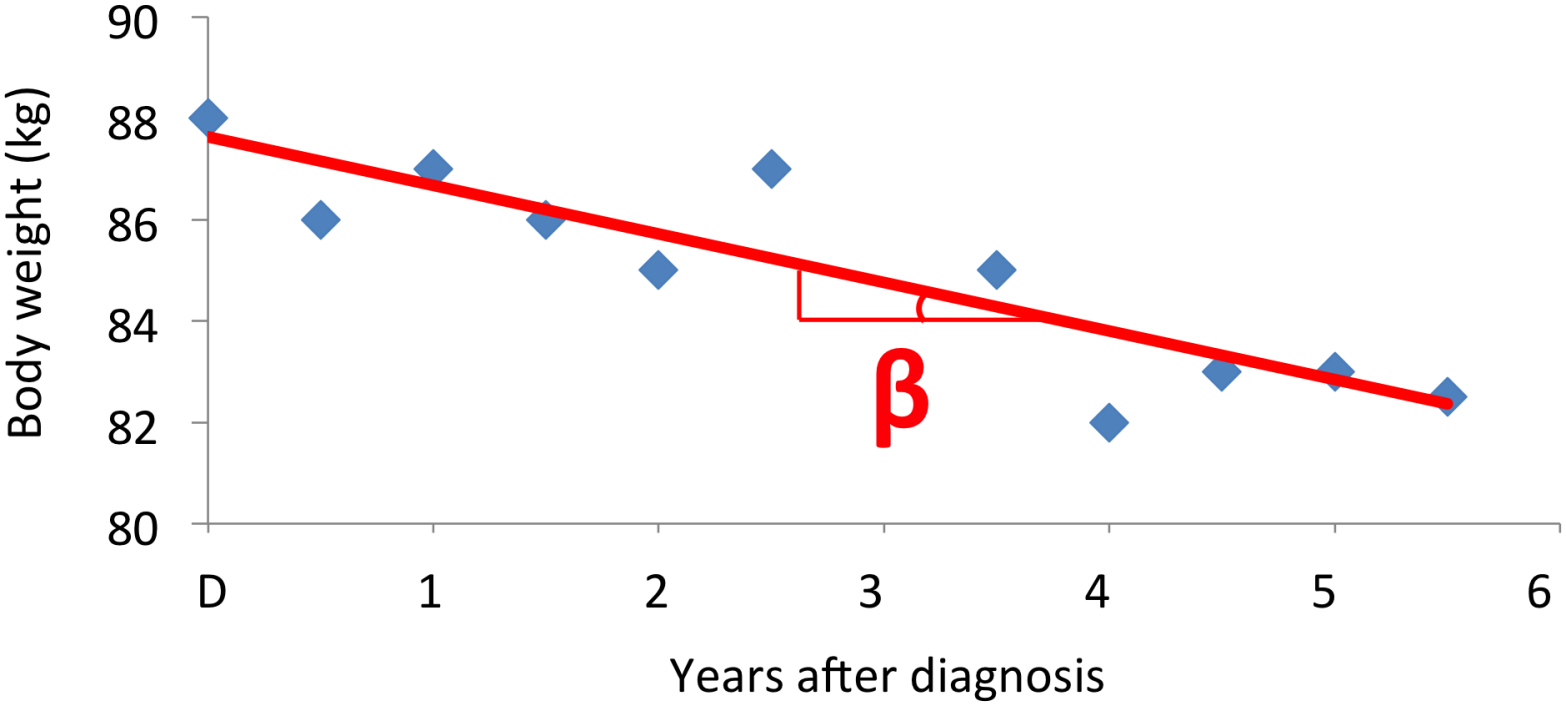
Weight change. This is an example of weight monitoring in one patient. For each patient weight change was modeled as a regression line through all the recorded weights. The exposure of interest in the present cohort study was the slope (the β coefficient) of this regression line. The exposure is a continuous variable that denotes the average yearly weight change (kg/year).

The 6-year DCGP intervention is referred to as the ‘monitoring period’ in the present cohort study. During this period the median number of weight registrations was 13 per patient, and the median time between consultations was 106 days. Weight change as defined in Fig 3 was the exposure of interest in the multivariable analyses.

### Definition of outcome

Information on all-cause mortality, cardiovascular mortality, and cardiovascular morbidity was obtained from the Danish Civil Registration System, the Danish National Patient Register, and the Danish Register of Causes of Death [17–19]. The patients were followed up in these registers until January 1^st^ 2009: a total of 13 years after the end of the monitoring period. Cardiovascular mortality was defined as fatal myocardial infarction (ICD-10: I20-I25 or I50), fatal stroke (I60-I69 or G45), fatal peripheral vascular disease (I70.2 or E10.5 or E11.5 or E12.5 or E13.5 or E14.5), or sudden death (R96-R99). Cardiovascular morbidity was defined as a fatal or non-fatal incidence of myocardial infarction, stroke, or peripheral vascular disease, as defined above.

### Definition of intention for weight change

The patients were categorized in four groups according to their intentions for weight change. The categorization is hierarchal, starting with no. 1:

1. *Aberrant weight pattern* (n = 5): The patient had a goal of weight gain at any time (n = 1), or a weight loss rate of > 20 kg/year between the two last check-ups (equivalent to 5 kg in 3 months), but did not have a goal of weight loss for that period (n = 4).
2. *Intention to lose weight* (n = 209): The patient had at least 3 recorded goals of weight loss, and at least one of these goals was recorded in one of the 3 last check-ups.
3. *Intention to maintain weight* (n = 210): The patient had at least one recorded goal of maintaining weight (and up to two recorded goals of weight loss).
4. *Intention for weight change not well-described* (n = 20): The patient had no recorded goals of maintaining weight (but up to two recorded goals of weight loss).

### Definition of potential confounders

Height was measured at diagnosis and weight was measured approximately every third month (without shoes) by the GP with the scales available in the clinic. BMI: baseline value (continuous). Smoking: self-reported at diagnosis and at the end of the monitoring period. Change in smoking status was used as a covariate (categorical: never-smoker, stable smoker, stable ex-smoker, quitter, or starter in the monitoring period). Physical activity: leisure time physical activity was self-reported at diagnosis and at the end of the monitoring period. Change in physical activity during the monitoring period was used as a covariate (categorical: remained sedentary, became sedentary, remained active, or became active). Education: <10 years of school or ≥10 years. Medication: start of antiglycemic medication in the monitoring period (categorical: methformin, sulfonylurea, combination of two or more oral antiglycemic medications, insulin, or none) [20]. The Charlson comorbidity index: score during the monitoring period (continuous) [21,22].

The presence of microvascular disease was evaluated at inclusion and yearly thereafter during the monitoring period by the GP (neuropathy, diabetic foot), by lab testing (nefropathy), and by an ophthalmologist (retinopathy) [16]. In this study microvascular disease was defined as the presence of one or more of these complications. Macrovascular disease was cardiocvscular morbidity as defined in the outcomes mentioned above.

### Statistical methods

The association between weight change and mortality/morbidity was analyzed with the hazard ratio (HR) from a Cox regression model, adjusted for age, sex, education, BMI at diagnosis, change in smoking, change in physical activity, change in medication, and the Charlson comorbidity 6-year score. The analysis was designed a priori to minimize bias from pathological weight loss. We assumed that weight loss among patients with the ‘intention to lose weight’ was less likely to be caused by a pathological process than weight loss among patients with the ‘intention to maintain weight’ [11,12,14,15]. Therefore, the main multivariable analysis was stratified in two separate analyses; one for each category of intention. Thus, mortality/morbidity attributable to weight change in these two categories of intention was not compared but the risk was estimated separately for each category.

The main model included only covariates with an anticipated causal effect on both the exposure (weight change over 6 years) and the outcomes. Intermediate variables like the mean HbA_1c_ in the monitoring period and the blood pressure, or triglyceride level at diagnosis, were included in additional sensitivity analyses to further adjust for disease severity. Other sensitivity analysis with a more narrow definition of the ‘intention to lose weight’ (6 or more recorded goals of weight loss, with at least one of these goals recorded in one of the 3 last check-ups), or for subgroups of patients with the ‘intention to lose weight’ further stratified on BMI (<30 vs. BMI≥30), microalbuminuria (≥15 vs. <15 mg/L), or established macrovascular disease (+/-) at diagnosis, were planned and performed. A post hoc sensitivity analysis adjusting for use of acetylcholine esterase inhibitors or angiotensine receptor blockers was made. No patients used glitazones and only 8 patients used statins at year 6. Therefore, no sensitivity analyses were made regarding use of these medications. There were no data available on use of acetylsalicylic acid.

As we expected most patients with pathological weight loss in the monitoring period to die within the first two years of the follow-up, the HR for the outcomes were analyzed separately for this period (year 6–8) and for the subsequent 11 years period (year 8–19). We regarded an association between weight loss in the monitoring period and mortality in the latter 11 years of follow-up (year 8–19), as being less affected by potential confounding from pathological weight loss. As we did not expect the associations to be linear, we modeled the relations by restricted cubic splines [23]. Only patients who were not registered as having cardiovascular morbidity (as defined for outcomes) by the end of the monitoring period were included in the analyses relating weight changes to cardiovascular morbidity. For 62 patients (14%), information on one or more of the covariates was missing, and they were omitted from the analyses. There was no loss to follow-up. All statistical analyses were conducted using SAS statistical software, version 9.2.

### Ethics Statement

This study was approved by the research ethics committee of Copenhagen and Frederiksberg (V.100.869/87). All patients gave oral informed consent to their GP, and this was recorded in the study files. Written informed consent was not customary in Denmark when the DCGP study was initiated, and the procedure used was approved by the research ethics committee.

## Results

Altogether 209 patients had the ‘intention to lose weight’ while 210 patients had the ‘intention to maintain weight’. Their characteristics at diabetes diagnosis and after 6 years of structured care (interquartile range 5.7–6.3 years) are described in Table 1. It is worthy of note that patients with the ‘intention to lose weight’ and patients with the ‘intention to maintain weight’ were not compared in the multivariable analyses. On the contrary, the main analysis was stratified by ‘intention to lose weight’ or ‘intention to maintain weight’.

**Table 1:** Characteristics of overweight diabetes patients with a well described aim for weight change during the 6 years weight monitoring period.

	Patients with the ‘intention to lose weight’ (n = 209)			Patients with the ‘intention to maintain weight’ (n = 210)		
	Value at diagnosis	Value after 6 years	Change	Value at diagnosis	Value after 6 years	Change
Male sex	103 (49)			109 (52)		
≥ 10 yrs of school	55 (27)			32 (16)		
Age, years	58.8±9.6	64.6±9.6	5.6±0.9	65.4±10.7	70.9±10.7	5.6±0.8
Weight, kg	91.6±16.1	88.5±15.7	-2.6±6.3	84.0±13.3	78.8±13.3	-5.1±6.9
BMI, kg/m^2^	32.6±4.7	31.6±4.8	-0.9±2.2	30.3±4.0	28.3±4.0	-1.8±2.6
Current smoking	73 (35)	61 (32)		72 (35)	60 (34)	
Fasting plasma glucose, mmol/l	14.0±4.0	8.8±2.9	-5.0±4.7	14.6±6.1	8.7±4.0	-5.8±7.4
HgbA_1c_, fract.	10.2±2.1	8.7±1.4	-1.5±2.2	10.1±2.0	8.7±1.6	-1.3±2.3
Systolic blood pressure, mmHg	150±22	147±21	-3.3±21.2	151±22	147±20	-2.2±24.8
Diastolic blood pressure, mmHg	88±10	84±10	-3.6±10.9	86±11	82±9	-3.5±11.4
Fasting triglycerides, mmol/l	2.9±3.0	2.2±1.4	-0.6±2.7	2.7±2.4	2.3±3.5	-0.5±3.1
Total cholesterols, mmol/l	6.5±1.5	6.1±1.1	-0.3±1.4	6.5±1.5	6.1±1.7	-0.4±1.4
Serum creatinine, μmol/l	88±14	91±19	3.1±15.8	94±20	105±73	10.8±61.3
GFR, mL/min/1,73 m^2^	73.8±13.9	68.8±15.4	-4.7±11.4	67.0±16.3	62.2±18.2	-4.8±13.1
Microvascular disease	97 (49)	108 (58)		121 (60)	110 (63)	
Macrovascular disease	52 (26)	57 (30)		61 (30)	80 (44)	
Statins	0 (0)	5 (3)		0 (0)	3 (2)	
ACE-inhibitors or A2 agonists	6 (3)	44 (22)		5 (2)	34 (17)	
Severe hypoglycaemia						
since diagnosis		7 (4)			5 (3)	
Dyslipidemia^a^	187 (92)	176 (94)		191 (94)	150 (86)	
Hypertension^b^	154 (74)	141 (71)		174 (83)	151 (77)	
Antidiabetic medicine						
Insulin		14 (7)			23 (12)	
Metformin		26 (13)			15 (8)	
Sulfolynurea		66 (33)			72 (37)	
Metf+Sulf		35 (18)			30 (15)	
Diet alone		58 (29)			56 (29)	

Values are numbers (%) or means ±SD. Change values are 6-year values minus baseline values.

^a^ Dyslipidemia is defined here as triglycerides ≥ 1,7 mmol/L and/or total cholesterol ≥ 5,0 mmol/L.

^b^ Hypertension is defined as systolic blood pressure ≥ 160 mmHg or diastolic ≥ 90 mmHg or use of blood pressure lowering medication.

The results from the analyses of the association between weight change and the outcomes over the 13-year follow-up period are presented in Table 2. In the unstratified analysis, during the entire follow-up period, and including all 444 patients regardless of their weight change intentions, a mean weight loss of 1 kg of per year was associated with an increase in all-cause mortality rate of 1.18 (95% CI 1.05–1.33, P<0.01), while weight change was not associated with cardiovascular mortality or morbidity. The increased risk was higher in the first two years compared with the remaining 11 years of the follow-up period for both all-cause mortality and cardiovascular mortality. Similar results were found in the stratified analysis of patients with the ‘intention to maintain weight’.

**Table 2:** Multivariable analyses of 13 years mortality and morbidity risk attributable to 1 kg of weight loss per year during a monitoring period in overweight patients with type 2 diabetes–main analyses.

	All patients		Patients with the ‘intention to lose weight’		Patients with the ‘intention to maintain weight’	
	HR (95% CI)	P	HR (95% CI)	P	HR (95% CI)	P
All-cause mortality	[217/379]		[92/191]		[125/188]	
First 2 years ^a^	1.42 (1.12–1.80)	<0.01	1.30 (0.80–2.14)	0.29	1.52 (1.14–2.03)	<0.01
After 2 years ^b^	1.13 (0.99–1.29)	0.06	1.18 (0.93–1.50)	0.16	1.13 (0.96–1.35)	0.15
Difference ^c^		0.10		0.72		0.05
Full follow-up period ^d^	1.18 (1.05–1.33)	<0.01	1.20 (0.97–1.50)	0.10	1.21 (1.03–1.41)	0.02
Cardiovascular mortality	[136/378]		[49/191]		[87/187]	
First 2 years	1.42 (1.09–1.86)	<0.01	1.39 (0.80–2.44)	0.25	1.50 (1.11–2.04)	<0.01
After 2 years	1.04 (0.88–1.23)	0.64	1.22 (0.86–1.72)	0.26	0.99 (0.81–1.21)	0.91
Difference		0.04		0.68		0.02
Full follow-up period	1.12 (0.97–1.30)	0.12	1.26 (0.93–1.72)	0.14	1.10 (0.92–1.30)	0.31
Cardiovascular morbidity	[132/310]		[55/161]		[77/149]	
First 2 years	1.26 (0.92–1.73)	0.16	1.35 (0.63–2.89)	0.44	1.22 (0.83–1.81)	0.32
After 2 years	0.95 (0.80–1.13)	0.56	1.02 (0.74–1.40)	0.92	0.89 (0.70–1.12)	0.30
Difference		0.12		0.50		0.15
Full follow-up period	1.01 (0.86–1.18)	0.93	1.06 (0.79–1.42)	0.71	0.95 (0.77–1.17)	0.64

Values are [number of events/numbers of observations used] and HR (95% confidence intervals).

^a^ HR for mortality in the first 2 years of follow-up.

^b^ HR for mortality in the remaining 11 years of follow-up (‘After 2 years’).

^c^ Difference in HR between the first 2 years and the remaining 11 years of follow-up.

^d^ The total follow-up period was 13 years. See the study timeline in Fig 2.

**Fig 4:**
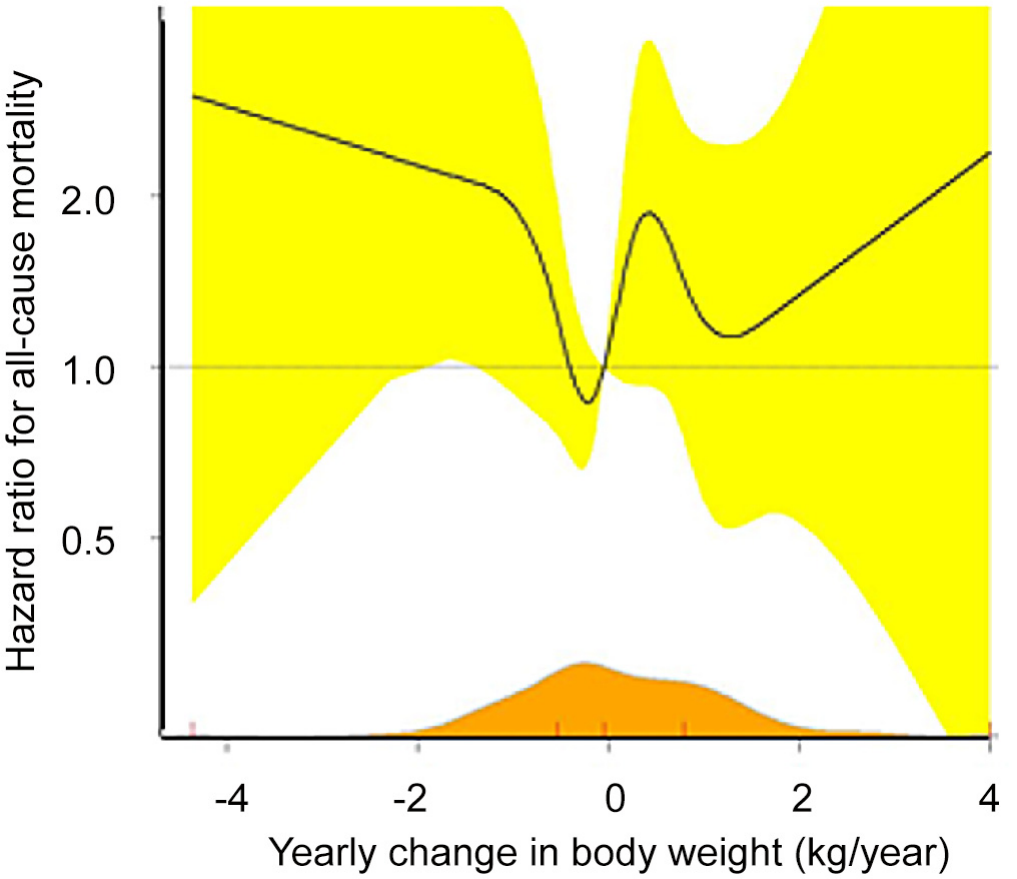
Weight change and mortality among overweight diabetes patients with an ‘intention to lose weight’. The figure demonstrates the association between the yearly weight change during the 6-year monitoring period after the diagnosis of type 2 diabetes and the subsequent 13-years HR for all-cause mortality. The y-axis is logarithmic. Black line: cubic spline estimate, 6 data driven nodes. Yellow: 95% confidence intervals. Orange: the distribution of the patient material. Red marks: median, interquartile range and min/max.

The main analysis in the study was of patients with an ‘intention to lose weight’. Among these overweight patients aiming to lose weight there was no difference in mortality rate attributable to weight loss between the first two years and the rest of the follow-up period (P = 0.72), and there was a trend toward a linear association between weight change and increased all-cause mortality 1.20 (95% CI 0.97–1.50, P = 0.10). In Fig 4 the adjusted HR for all-cause mortality is depicted as a spline function of the yearly weight change rate (the β-coefficient from Fig 3). Zero on the x-axis signifies that the weight on average was maintained throughout the monitoring period, whereas a negative value denotes a general weight loss. Fig 4 suggests a V-like association between weight change and all-cause mortality suggesting a trend towards the lowest mortality in those maintaining their weight despite their intention to lose weight.

### Sensitivity analyses

For patients with an ‘intention to lose weight’ we performed a range of planned sensitivity analyses to test the robustness of the association between weight change and the 13-year all-cause mortality rate. The inclusion of the mean HbA_1c_ in the monitoring period, the triglyceride level, the diastolic or systolic blood pressure at diagnosis as covariates did not substantially change the estimates. A more narrow definition of ‘intention to lose weight’ (as defined in Methods) also gave essentially similar results. Analyses stratified by BMI indicate that associations between weight loss and all-cause mortality may be driven by patients with a BMI≥30 (Table 3). In these obese patients, intentional weight loss was associated with an increased mortality (HR 1.37, 95% CI 1.01–1.84, P = 0.04), whereas in overweight patients with BMI<30 weight loss was not associated with mortality (0.65, 0.37–1.13, P = 0.12). In patients with microalbuminuria at diagnosis, intentional weight loss was associated with increased all-cause mortality (HR 1.90, 1.03–3.48, P = 0.04), while in patients without established cardiovascular disease or microalbuminuria at baseline, intentional weight loss was not associated with mortality (HR 1.00, 0.59–1.71, P = 0.99). Furthermore, a post hoc analysis of patients who became or remained physically active did not indicate a protective effect of intentional weight loss (HR 1.07, 0.83–1.37, P = 0.61). Adjustment for the use of acetylcholine esterase inhibitors or angiotensin receptor blockers did not change the association (HR 1.20, 0.96–1.50, P = 0.10).

**Table 3:** Multivariable analyses of 13 years mortality and morbidity risk attributable to 1 kg of weight loss per year during a monitoring period in overweight patients with type 2 diabetes–sensitivity analyses.

	All patients with BMI<30 kg/m^2^		All patients with BMI>30 kg/m^2^		BMI<30 kg/m^2^ and ‘intention to lose weight’		BMI>30 kg/m^2^ and ‘intention to lose weight’	
	HR (95% CI)	P	HR (95% CI)	P	HR (95% CI)	P	HR (95% CI)	P
All-cause mortality	[102/167]		[115/212]		[36/63]		[56/128]	
First 2 years ^a^	1.55 (0.90–2.66)	0.11	1.47 (1.08–2.00)	0.01	0.61 (0.12–3.19)	0.55	1.48 (0.85–2.57)	0.17
After 2 years ^b^	0.92 (0.69–1.22)	0.55	1.17 (1.01–1.36)	0.04	0.65 (0.36–1.16)	0.14	1.34 (0.97–1.85)	0.08
Difference ^c^		0.07		0.18		0.93		0.74
Full follow-up period ^d^	1.01 (0.77–1.32)	0.97	1.22 (1.06–1.40)	0.01	0.65 (0.37–1.13)	0.12	1.37 (1.01–1.84)	0.04
Cardiovascular mortality	[60/166]		[76/212]		[19/63]		[30/128]	
First 2 years	1.73 (0.93–3.22)	0.08	1.43 (1.04–1.99)	0.03	0.88 (0.12–6.75)	0.91	1.19 (0.59–2.41)	0.63
After 2 years	1.00 (0.68–1.46)	0.99	1.05 (0.85–1.29)	0.66	0.87 (0.34–2.22)	0.77	0.95 (0.60–1.51)	0.84
Difference		0.11		0.10		0.99		0.58
Full follow-up period	0.92 (0.64–1.33)	0.65	1.10 (0.92–1.31)	0.31	0.92 (0.37–2.29)	0.86	1.08 (0.70–1.66)	0.74
Cardiovascular morbidity	[58/135]		[74/175]		[25/55]		[30/106]	
First 2 years	0.91 (0.43–1.92)	0.80	1.39 (0.93–2.06)	0.11	1.84 (0.42–7.98)	0.42	1.48 (0.38–5.81)	0.58
After 2 years	0.82 (0.57–1.19)	0.29	1.01 (0.82–1.26)	0.93	1.17 (0.57–2.42)	0.67	1.04 (0.67–1.62)	0.85
Difference		0.80		0.15		0.57		0.63
Full follow-up period	0.83 (0.59–1.18)	0.31	1.08 (0.89–1.31)	0.46	1.25 (0.63–2.49)	0.52	1.07 (0.69–1.65)	0.76

Values are [number of events/numbers of observations used] and HR (95% confidence intervals).

^a^ HR for mortality in the first 2 years of follow-up.

^b^ HR for mortality in the remaining 11 years of follow-up (‘After 2 years’).

^c^ Difference in HR between the first 2 years and the remaining 11 years of follow-up.

^d^ The total follow-up period was 13 years. See the study timeline in Fig 2.

## Discussion

### Principal findings

In this population-based cohort study of newly diagnosed overweight patients with type 2 diabetes, a therapeutic intentional weight loss, supervised over 6 years by a medical doctor, was not associated with reduced mortality or cardiovascular morbidity in the following 13 years. Rather, the lowest mortality was seen among those patients who maintained their weight during the period.

Among all 444 overweight patients included in the study, weight loss (regardless of intention) was an independent risk factor for all-cause mortality, but this association was primarily driven by unintentional weight loss in patients with the ‘intention to maintain weight’. However, excess all-cause mortality was also related to intentional weight loss among patients with BMI≥30 kg/m^2^ and among patients with microalbuminuria at diagnosis. In patients with BMI<30 kg/m^2^ there was a trend towards decreased mortality in relation to intentional weight loss, but this association was not significant. This could be a chance finding. Other possible explanations could be a higher degree of residual confounding from comorbidity among the obese patients or differences in weight loss methods between overweight and obese patients. Our finding that intentional weight loss was associated with increased mortality in patients with microalbuminuria at diagnosis could indicate that the weight loss in these patients was a consequence of disease severity, or that therapeutic weight loss was, in fact, detrimental in these patients. Nevertheless, intentional weight loss was not associated with survival in patients without established cardiovascular disease or microalbuminuria at baseline.

### Strengths and weaknesses of the study

There are several strengths to this study. For instance, the study was truly population based, the participants were prospectively monitored with numerous physical examinations, they were followed up in the nationwide Danish health registers [17–19], and there was no loss to follow-up. Also, the analyses were carefully designed to minimize confounding from wasting: 1) The very conservative definition of ‘intention to lose weight’ ensured that only patients persistently intending to lose weight were included in the main analysis; 2) The analyses were adjusted for incident comorbidity and changes in antidiabetic medication in the monitoring period [20]; 3) All patients with prior or incident cancer were excluded; 4) Multiple indicators for disease severity were included in the sensitivity analyses, but the results were robust; 5) The follow-up period was divided in two (the first two years and the remaining 11 years). The mortality rate attributable to weight loss did not differ between these two periods among patients with an ‘intention to lose weight’, indicating low bias from pathological weight loss. Still, it is a limitation that cannot be excluded that the results may be partly explained by residual confounding from pathological weight loss.

It is also a limitation that patients younger than 40 years were not included, as patients with diabetes, in most settings, are diagnosed earlier today than they were 25 years ago. In 1990, patients were commonly diagnosed with clinical diabetes, while most patients today are diagnosed by screening. Thus, the results can only be generalized to patients with clinical diabetes. The control group from the original trial was not included in the study, but since the study was randomized the participants we included are still representative of patients with incident diabetes in the general population 25 years ago. This is actually a strength because all the patients included received the intervention that later became the standard care for patients with type 2 diabetes in Denmark. However, risk factors for cardiovascular disease are treated much more aggressively today than they were 25 years ago, while lifestyle recommendations are largely unchanged.

There was considerable intra-individual variation in weight development, and a slope of a regression line may seem too simple a method to describe the individual weight change pattern. For instance, a patient with high compliance may lose 6 kilos fast and then maintain that weight for 6 years. This would result in a relatively flat slope compared to a patient who loses 1 kg a year, resulting in a steeper slope. Consequently, the patient with slow weight loss would get a higher numeric value for the exposure. Still, this method certainly better describes the general weight change than retrospective and self-reported weight changes that earlier studies of intentional weight loss in patients with type 2 diabetes have used [7,8,10,12]. The analysis was adjusted for baseline BMI, which to some degree correlates with fat mass [24], but we did not have access to advanced data on our participants’ body composition or the tissue composition of the weight loss.

### Strengths and weaknesses in relation to other studies

The present study is the first cohort study to examine intentional weight loss in patients with type 2 diabetes where the diagnosis was biochemically verified, intentions for weight change were prospectively described, and body weight was assessed by clinical examination. In contrast to most other observational studies in the field, our study does not compare mortality between those who lost weight intentionally, and those with stable weight and unknown intention or intention to maintain weight [14]. Gregg et al demonstrated that the mere ‘intention to lose weight’ in subjects both with and without diabetes was associated with reduced mortality regardless of whether they actually lost weight or not [12,25]. Intending to lose weight is most likely not in itself protective, but may be a good proxy for true explanatory factors like health literacy, compliance, and a healthy lifestyle in general. Thus, comparing weight loss among participants with diverse weight loss intentions may well bias the result towards a favorable effect of weight loss. The present study avoided this bias by exploring the correlation between weight change and the outcomes *within* the group of patients intending to lose weight. As weight change was a continuous variable in our study (Fig 3), there was no categorical control group in the multivariable analysis.

In a previous study of weight history among participants in the DCGP study we found an average weight loss after diagnosis in all age categories [26], which may suggest that weight loss is part of the natural history of diabetes, and that an average weight loss trend started at diabetes diagnosis. However, the individual variability was large, and the present study explored this variability.

Several studies have reported reduced mortality in obese patients with diabetes or established cardiovascular disease, compared with lean patients [27]. This ‘obesity paradox’ is seen in study designs exploring the association between a single measurement of weight/BMI and subsequent mortality. The phenomenon may well be an artifact coming from pre-existing illness (that results in pathological weight loss and higher mortality among lower BMI groups, making obesity appear protective) and collider stratification bias [27]. Our study does not explore a relation between weight/BMI and mortality, but analyzes implications of *weight change* over 6 years.

### Results in relation to other studies

Our main findings are in opposition to results from other observational studies in the field. For instance, Harrington’s meta-analysis of weight loss found that among unhealthy obese subjects (in this context BMI≥25–27) intentional weight loss was associated with reduced mortality [14]. This result was mainly based on studies of patients with type 2 diabetes [10,12]. However, as indicated above data quality and bias favoring weight loss may pertain to these studies. Furthermore, our results are in line with the Look AHEAD trial that found no cardiovascular protective effect of an intensive lifestyle intervention that resulted in substantial weight loss. However, it is not straightforward to interpret a randomized trial with a complex ‘intensive lifestyle intervention’, as both diet and physical activity by themselves may affect both the weight loss and the disease outcomes [28–32]. Thus, it is not possible to conclude whether the effect of the lifestyle-based weight loss therapy on the hard outcomes was caused by the weight loss per se, or by the change in the composition of the diet, the increase in exercise, or by other effects of the intervention. Unlike Look AHEAD, the present study is an analysis of the weight change itself.

### Conclusions

In conclusion, our results do not support our hypothesis that overweight patients with type 2 diabetes who undergo a successful therapeutic intentional weight loss, supervised by a medical doctor, experience reduced mortality or cardiovascular morbidity. Rather, the patients who maintained their weight had the best prognosis. The findings in this population based cohort study are in line with those of the Look AHEAD trial.
